# Author Correction: Application of Mendelian randomization to explore the causal role of the human gut microbiome in colorectal cancer

**DOI:** 10.1038/s41598-023-38170-1

**Published:** 2023-07-26

**Authors:** Charlie Hatcher, George Richenberg, Samuel Waterson, Long H. Nguyen, Amit D. Joshi, Robert Carreras-Torres, Victor Moreno, Andrew T. Chan, Marc Gunter, Yi Lin, Conghui Qu, Mingyang Song, Graham Casey, Jane C. Figueiredo, Stephen B. Gruber, Jochen Hampe, Heather Hampel, Mark A. Jenkins, Temitope O. Keku, Ulrike Peters, Catherine M. Tangen, Anna H. Wu, David A. Hughes, Malte C. Rühlemann, Jeroen Raes, Nicholas J. Timpson, Kaitlin H. Wade

**Affiliations:** 1 MRC Integrative Epidemiology Unit, University of Bristol, Oakfield House, Oakfield Grove, Bristol, BS8 2BN UK; 2 Population Health Sciences, Bristol Medical School, University of Bristol, Bristol, BS8 2BN UK; 3 North Bristol NHS Trust, Bristol, BS10 5NB UK; 4 Massachusetts General Hospital, Boston, MA 02114 USA; 5 Colorectal Cancer Group, ONCOBELL Program, Bellvitge Biomedical Research Institute (IDIBELL), L’Hospitalet de Llobregat, 08908 Barcelona, Spain; 6 Digestive Diseases and Microbiota Group, Girona Biomedical Research Institute (IDIBGI), 17190 Salt, Girona, Spain; 7 Oncology Data Analytics Program, Catalan Institute of Oncology (ICO), Hospitalet de Llobregat, Barcelona, Spain; 8 Biomedical Research Centre Network for Epidemiology and Public Health (CIBERESP), Madrid, Spain; 9 Department of Clinical Sciences, Universitat de Barcelona Institute of Complex Systems (UBICS), Faculty of Medicine and Health Sciences, University of Barcelona, Barcelona, Spain; 10 International Agency for Research on Cancer, 150 Cours Albert Thomas, 69372 Lyon, CEDEX 08, France; 11 Fred Hutchinson Cancer Research Center, 1100 Fairview Ave. N., Seattle, WA 98109 USA; 12 Public Health Sciences Division, Fred Hutchinson Cancer Center, Seattle, WA USA; 13 Department of Epidemiology, Harvard T.H. Chan School of Public Health, Boston, MA 02115 USA; 14 Division of Gastroenterology, Clinical and Translational Epidemiology Unit, Massachusetts General Hospital, Harvard Medical School, Boston, MA 02115 USA; 15 Department of Nutrition, Harvard T.H. Chan School of Public Health, Boston, MA 02115 USA; 16 Center for Public Health Genomics, University of Virginia, Charlottesville, VA USA; 17 Department of Medicine, Samuel Oschin Comprehensive Cancer Institute, Cedars-Sinai Medical Center, Los Angeles, CA USA; 18 Department of Preventive Medicine, USC Norris Comprehensive Cancer Center, Keck School of Medicine, University of Southern California, Los Angeles, CA USA; 19 Department of Medicine I, University Hospital Dresden, Technische Universität Dresden (TU Dresden), Dresden, Germany; 20 Division of Human Genetics, Department of Internal Medicine, The Ohio State University Comprehensive Cancer Center, Columbus, OH USA; 21 Centre for Epidemiology and Biostatistics, Melbourne School of Population and Global Health, The University of Melbourne, Melbourne, VIC Australia; 22 Center for Gastrointestinal Biology and Disease, University of North Carolina, Chapel Hill, NC USA; 23 SWOG Statistical Center, Fred Hutchinson Cancer Research Center, Seattle, WA USA; 24 Preventative Medicine, University of Southern California, Los Angeles, CA USA; 25 Institute of Clinical Molecular Biology, Christian Albrechts University of Kiel, Kiel, Germany; 26 Department of Microbiology and Immunology, Rega Instituut, KU Leuven, University of Leuven, Leuven, Belgium; 27 Center for Microbiology, VIB, Leuven, Belgium

Correction to:* Scientifc Reports* https://doi.org/10.1038/s41598-023-31840-0, published online 12 April 2023

The original version of this Article contained errors. The colocalisation analysis parameters using the ‘coloc’ package detailing the outcome dataset type were incorrectly specified and default prior probability was erroneously changed from 1e−05 to 1e−06.

As a result, in the Results section, under the subheading ‘Sensitivity analyses’,

“Therefore, focusing on the difference between the tested hypotheses (see Methods), colocalisation results provided evidence that neither *G. Bifidobacterium* nor *G. unclassified, O. Bacteroidales* were likely to share a causal variant with overall or site-specific CRC (Table 2). Regional association plots confirmed findings from genetic colocalisation for *G. unclassified, O. Bacteroidales* (Fig. 3; Supplementary Fig. 1). However, despite little evidence for genetic colocalisation for *G. Bifidobacterium* with overall and site-specific CRC, regional association plots suggest the genomic region surrounding the rs4988235 SNP is important in both abundance of *G. Bifidobacterium* and CRC risk (Fig. 3; Supplementary Fig. 2).”

now reads:

“Therefore, focusing on the difference between the tested hypotheses (see Methods), colocalisation results provided little evidence that *G. unclassified, O. Bacteroidales* was likely to share a causal variant with overall or site-specific CRC (Table 2). Regional association plots confirmed findings from genetic colocalisation for *G. unclassified, O. Bacteroidales* (Fig. 3; Supplementary Fig. 1). Whilst there was evidence for genetic colocalisation for *G. Bifidobacterium* with overall (with a posterior probability of 0.87) CRC risk and, to a lower extent, with site-specific CRC risk (posterior probabilities ranged from 0.05–0.72), regional association plots suggest the wider genomic region surrounding the rs4988235 SNP is important in both abundance of *G. Bifidobacterium* and CRC risk (Fig. 3; Supplementary Fig. 2).”

Also, in the Discussion section,

“It is worth noting that, whilst limited by power, our colocalisation analyses provided little evidence to suggest that either of these microbial traits (i.e., *G. Bifidobacterium* and *G. unclassified, O. Bacteroidales*) shared causal variants with CRC, which is a necessary (but not a sufficient) criterion for causality. Therefore, further analyses with a much larger quantity of microbiome-related SNPs, larger GWASs from which these SNPs are discovered and importantly replicated, plus a better understanding of the relationships between host genetic variation and the gut microbiome are required to provide conclusive evidence in this context.”

now reads:

“It is worth noting that, whilst limited by power, our colocalisation analyses provided some evidence to suggest that *G. Bifidobacterium* (but not *G. unclassified, O. Bacteroidales*) shared causal variants with CRC, which is a necessary (but not a sufficient) criterion for causality. Therefore, further analyses with a much larger quantity of microbiome-related SNPs, larger GWASs from which these SNPs are discovered and importantly replicated, plus a better understanding of the relationships between host genetic variation and the gut microbiome and comprehensive sensitivity analyses that we present here are required to provide conclusive evidence in this context.”

Further, in the Materials and methods section, under the subheading ‘Sensitivity analyses’,

“Colocalisation analyses were conducted using the ‘coloc’ R package with default parameters^40^.”

now reads:

“Colocalisation analyses were conducted using the ‘coloc’ R package with default parameters (i.e., with the prior probabilities of the SNP being associated with the exposure, the outcome or both traits being specified as 1 × 10^−04^, 1 × 10^−04^ and 1 × 10^−05^, respectively^40^), additionally specifying that the exposures (i.e., microbial traits) were continuous and that the outcome (i.e., CRC risk) was binary (with either the ‘quant’ or ‘cc’ option, respectively, specified as the type of data).”

Moreover, in Table 2, all the values were incorrect. The original Table 2 appears below.

**Table 2:** Posterior probabilities relating to associations between microbial traits and both overall and site-specific colorectal cancer.

Microbial trait and CRC risk	H0	H1	H2	H3	H4
*G. Bifidobacterium* (AB) and overall CRC	0.86	0.11	0.03	0.004	0.0001
*G. Bifidobacterium* (AB) and distal colon cancer risk	0.85	0.11	0.04	0.01	0.0001
*G. Bifidobacterium* (AB) and proximal colon cancer risk	0.85	0.11	0.04	0.01	0.0001
*G. Bifidobacterium* (AB) and colon cancer risk	0.86	0.11	0.03	0.004	0.0001
*G. Bifidobacterium* (AB) and rectal cancer risk	0.85	0.11	0.04	0.01	0.0001
*G. unclassified, O. Bacteroidales* (P/A) and overall CRC	0.78	0.16	0.05	0.01	0.0002
*G. unclassified, O. Bacteroidales* (P/A) and proximal colon cancer risk	0.79	0.14	0.06	0.01	0.0002
*G. unclassified, O. Bacteroidales* (P/A) and colon cancer risk	0.80	0.14	0.04	0.01	0.0001
*G. unclassified, O. Bacteroidales* (P/A) and rectal cancer risk	0.79	0.14	0.06	0.01	0.0002

AB = abundance; CRC = colorectal cancer; P/A = presence versus absence. Letters in the microbial trait name represent the taxon classification level from which that microbial trait was observed, with “*G*” and “*O*” representing “genus” and “order”, respectively. All microbial traits that were not confidently classified at the genus level were organised into unclassified groups within higher classification levels (represented by “unclassified”). *H*0: Neither trait has a genetic association in the region, H1: Only the first trait (i.e., the microbial trait) has a genetic association in the region, *H*2; Only the second trait (i.e., breast cancer risk) has a genetic association in the region, *H*3: Both traits are associated but have different causal variants and *H*4: Both traits are associated and have the same causal variant.

Finally, the Supplementary Information 2 file contained an error, where the column headings ‘EA’ and ‘OA’ in Supplementary Table 2 were inadvertently reversed (except for *G. Bifidobacterium*).

The original Article and its accompanying Supplementary Information 2 file have been corrected.

