# Supplementary material for: Application of Mendelian randomization to explore the causal role of the human gut microbiome in colorectal cancer
Source: Sci Rep. 2023 Apr 12;13:5968. doi: 10.1038/s41598-023-31840-0 (PMC10097673; doi:10.1038/s41598-023-31840-0)
Supplement: Supplementary file 3 — Supplementary Information 3. [file 41598_2023_31840_MOESM3_ESM.docx]

**STROBE-MR checklist of recommended items to address in reports of Mendelian randomization studies**^1^ ^2^

| **Item No.** | **Section** | **Checklist item** | **Page No.** | **Relevant text from manuscript** |
| --- | --- | --- | --- | --- |
| 1 | **TITLE and ABSTRACT** | Indicate Mendelian randomization (MR) as the study’s design in the title and/or the abstract if that is a main purpose of the study | 1-3 | Title is “Application of Mendelian randomization to explore the causal role of the human gut microbiome in colorectal cancer” and abstract details the purpose and methodology of the study. |
|  | **INTRODUCTION** |  | 4-5 |  |
| 2 | **Background** | Explain the scientific background and rationale for the reported study. What is the exposure? Is a potential causal relationship between exposure and outcome plausible? Justify why MR is a helpful method to address the study question | 4-5 | Rationale for the study is detailed in the first three paragraphs; the exposure is detailed in the second paragraph; and the potential causal relationship is in the second paragraph; and the justification of using MR is in the third and final paragraph. |
| 3 | **Objectives** | State specific objectives clearly, including pre-specified causal hypotheses (if any). State that MR is a method that, under specific assumptions, intends to estimate causal effects | 5 | Objectives clearly outlined in the last paragraph of the introduction. |
|  | **METHODS** |  | 15-22 |  |
| 4 | **Study design and data sources** | Present key elements of the study design early in the article. Consider including a table listing sources of data for all phases of the study. For each data source contributing to the analysis, describe the following: | 15-17 | A summary of the study design is provided on page 15 with the data sources overview being provided on pages 15-17. |
|  | a) | Setting: Describe the study design and the underlying population, if possible. Describe the setting, locations, and relevant dates, including periods of recruitment, exposure, follow-up, and data collection, when available. | 15-17 | A summary of the study design is provided on page 15 with the data sources overview being provided on pages 15-17. |
|  | b) | Participants: Give the eligibility criteria, and the sources and methods of selection of participants. Report the sample size, and whether any power or sample size calculations were carried out prior to the main analysis | 15-17 | A summary of the study design is provided on page 16, the data sources are detailed on page 15-17 (which includes an overview of the participants, criteria for inclusion and selection of studies and participants). Power calculations were not carried out before the analysis. Numbers of cases and controls of the outcome also provided in Supplementary Table S1. |
|  | c) | Describe measurement, quality control and selection of genetic variants | 15-17 | Genetic measurements and selection of instruments are provided. |
|  | d) | For each exposure, outcome, and other relevant variables, describe methods of assessment and diagnostic criteria for diseases | 15-17 | Measurements of the exposures and outcomes are detailed throughout these pages. |
|  | e) | Provide details of ethics committee approval and participant informed consent, if relevant | 15 | Information on ethical approval is provided on page 15. |
| 5 | **Assumptions** | Explicitly state the three core IV assumptions for the main analysis (relevance, independence and exclusion restriction) as well assumptions for any additional or sensitivity analysis | 19 | Assumptions are provided on page 19 as well as in Figure 1. |
| 6 | **Statistical methods: main analysis** | Describe statistical methods and statistics used | 17-19 | A clear description of all analyses are provided. |
|  | a) | Describe how quantitative variables were handled in the analyses (i.e., scale, units, model) | 17-19 | Units and transformations are provided for all quantitative analyses, as well as how these estimates can be interpreted. |
|  | b) | Describe how genetic variants were handled in the analyses and, if applicable, how their weights were selected | 17-19 | Weights are not provided (as this is a two-sample MR) but the methodology for MR effect estimation are provided (i.e., the Wald ratio for main analyses). |
|  | c) | Describe the MR estimator (e.g. two-stage least squares, Wald ratio) and related statistics. Detail the included covariates and, in case of two-sample MR, whether the same covariate set was used for adjustment in the two samples | 17-19 | The methodology for MR effect estimation are provided (i.e., the Wald ratio and the inverse variance weighted method) as well as how the F-statistics and R-squared are calculated. |
|  | d) | Explain how missing data were addressed | NA | NA |
|  | e) | If applicable, indicate how multiple testing was addressed | 22 | Multiple testing correction details for main analyses are provided and justified on page 22. |
| 7 | **Assessment of assumptions** | Describe any methods or prior knowledge used to assess the assumptions or justify their validity | 18 | F-statistic and R-squared calculations are provided (relating to the “relevance” assumption). |
| 8 | **Sensitivity analyses and additional analyses** | Describe any sensitivity analyses or additional analyses performed (e.g. comparison of effect estimates from different approaches, independent replication, bias analytic techniques, validation of instruments, simulations) | 19-22 | All sensitivity analyses are described and justified. |
| 9 | **Software and pre-registration** |  |  |  |
|  | a) | Name statistical software and package(s), including version and settings used | 19-22 | Software for calculating the R-squared and those used for main and sensitivity analyses are provided. |
|  | b) | State whether the study protocol and details were pre-registered (as well as when and where) | NA | NA |
|  | **RESULTS** |  | 6-10 |  |
| 10 | **Descriptive data** |  |  |  |
|  | a) | Report the numbers of individuals at each stage of included studies and reasons for exclusion. Consider use of a flow diagram | 6; 15-17 | The numbers of individuals in the exposure and outcome dataset are presented in the first paragraph of the results and in the first three sections of the methods. |
|  | b) | Report summary statistics for phenotypic exposure(s), outcome(s), and other relevant variables (e.g. means, SDs, proportions) | NA | These are not provided by the individual genome-wide association studies. Numbers of cases and controls for the outcome are provided in Supplementary Table S1. |
|  | c) | If the data sources include meta-analyses of previous studies, provide the assessments of heterogeneity across these studies | NA | NA |
|  | d) | For two-sample MR:  i.  Provide justification of the similarity of the genetic variant-exposure associations between the exposure and outcome samples  ii.  Provide information on the number of individuals who overlap between the exposure and outcome studies | 6; 15-17 | Information about the study population provided in the methods (pages 15-17) and results (page 6). |
| 11 | **Main results** |  |  |  |
|  | a) | Report the associations between genetic variant and exposure, and between genetic variant and outcome, preferably on an interpretable scale | 6 | Also provided in Supplementary Table S2. |
|  | b) | Report MR estimates of the relationship between exposure and outcome, and the measures of uncertainty from the MR analysis, on an interpretable scale, such as odds ratio or relative risk per SD difference | 6-7 | Results provided for all exposures and outcomes in text and in Table 1, as well as Figure 2. |
|  | c) | If relevant, consider translating estimates of relative risk into absolute risk for a meaningful time period | NA | NA |
|  | d) | Consider plots to visualize results (e.g. forest plot, scatterplot of associations between genetic variants and outcome versus between genetic variants and exposure) | Figure 2 | Figure provides results from MR analyses. |
| 12 | **Assessment of assumptions** |  |  |  |
|  | a) | Report the assessment of the validity of the assumptions | 6-10 | F-statistics (assessing first “relevance” assumption) and sensitivity analyses (assessing third “exclusion restriction” assumption) provided. F-statistics also provided in Supplementary Table S2. |
|  | b) | Report any additional statistics (e.g., assessments of heterogeneity across genetic variants, such as *I^2^*, Q statistic or E-value) | 7-10 | Additional analyses assessing heterogeneity and robustness of results provided. |
| 13 | **Sensitivity analyses and additional analyses** |  |  |  |
|  | a) | Report any sensitivity analyses to assess the robustness of the main results to violations of the assumptions | 7-10 | Results from all sensitivity analyses provided as well as in Figures 3-4, Table 2 and Supplementary Tables S3-S15. |
|  | b) | Report results from other sensitivity analyses or additional analyses | NA | NA |
|  | c) | Report any assessment of direction of causal relationship (e.g., bidirectional MR) | 10 | Results from reverse MR provided as well as in Supplementary Table 16. |
|  | d) | When relevant, report and compare with estimates from non-MR analyses | NA | NA |
|  | e) | Consider additional plots to visualize results (e.g., leave-one-out analyses) | NA | NA |
|  | **DISCUSSION** |  | 11-14 |  |
| 14 | **Key results** | Summarize key results with reference to study objectives | 11-12 | Key results and interpretation provided. |
| 15 | **Limitations** | Discuss limitations of the study, taking into account the validity of the IV assumptions, other sources of potential bias, and imprecision. Discuss both direction and magnitude of any potential bias and any efforts to address them | 13-14 | All limitations discussed in detail. |
| 16 | **Interpretation** |  |  |  |
|  | a) | Meaning: Give a cautious overall interpretation of results in the context of their limitations and in comparison with other studies | 11-14 | Cautious overview of work provided. |
|  | b) | Mechanism: Discuss underlying biological mechanisms that could drive a potential causal relationship between the investigated exposure and the outcome, and whether the gene-environment equivalence assumption is reasonable. Use causal language carefully, clarifying that IV estimates may provide causal effects only under certain assumptions | 11-12 | Possible mechanisms described but given overall conclusion, this is not discussed in the conclusive statement. |
|  | c) | Clinical relevance: Discuss whether the results have clinical or public policy relevance, and to what extent they inform effect sizes of possible interventions | 14 | Possible clinical message described but given overall conclusion, this is not discussed in the conclusive statement. |
| 17 | **Generalizability** | Discuss the generalizability of the study results (a) to other populations, (b) across other exposure periods/timings, and (c) across other levels of exposure | 14 | Generalisability discussed in terms of population samples used in MR analyses. |
|  | **OTHER INFORMATION** |  |  |  |
| 18 | **Funding** | Describe sources of funding and the role of funders in the present study and, if applicable, sources of funding for the databases and original study or studies on which the present study is based | 27 | Funding statements and acknowledgements provided. |
| 19 | **Data and data sharing** | Provide the data used to perform all analyses or report where and how the data can be accessed, and reference these sources in the article. Provide the statistical code needed to reproduce the results in the article, or report whether the code is publicly accessible and if so, where | 27 | Data access and availability provided. |
| 20 | **Conflicts of Interest** | All authors should declare all potential conflicts of interest | 27 | Provided for those authors with conflicts of interest. |

This checklist is copyrighted by the Equator Network under the Creative Commons Attribution 3.0 Unported (CC BY 3.0) license.

1. Skrivankova VW, Richmond RC, Woolf BAR, Yarmolinsky J, Davies NM, Swanson SA, et al. Strengthening the Reporting of Observational Studies in Epidemiology using Mendelian Randomization (STROBE-MR) Statement. JAMA. 2021;under review.

2. Skrivankova VW, Richmond RC, Woolf BAR, Davies NM, Swanson SA, VanderWeele TJ, et al. Strengthening the Reporting of Observational Studies in Epidemiology using Mendelian Randomisation (STROBE-MR): Explanation and Elaboration. BMJ. 2021;375:n2233.
